# The Effect of Religious Dietary Cultures on Food Nitrogen and Phosphorus Footprints: A Case Study of India

**DOI:** 10.3390/nu13061926

**Published:** 2021-06-03

**Authors:** Aurup Ratan Dhar, Azusa Oita, Kazuyo Matsubae

**Affiliations:** 1 Graduate School of Environmental Studies, Tohoku University, 468-1 Aoba, Aramaki, Aoba-ku, Sendai 980-0845, Miyagi, Japan; kazuyo.matsubae.a2@tohoku.ac.jp; 2 Institute for Agro-Environmental Sciences, National Agriculture and Food Research Organization, 3-1-3, Kannondai, Tsukuba 305-8604, Ibaraki, Japan; a.oita@affrc.go.jp

**Keywords:** bottom-up approach, culture and religion, food consumption, nutrient management, religion-sensitive footprint methods

## Abstract

The excessive consumption of nitrogen (N) and phosphorus (P), two vital nutrients for living organisms, is associated with negative environmental and health impacts. While food production contributes to a large amount of N and P loss to the environment, very little N and P is consumed as food. Food habits are affected by multiple regulations, including the dietary restrictions and dictates of various religions. In this study, religion-sensitive N-Calculator and P-Calculator approaches were used to determine the impact of religious dietary culture on the food N and P footprints of India in the major religious communities. Using 2013 data, the food N footprint of Hindus, Muslims, Christians, and Buddhists was 10.70, 11.45, 11.47, and 7.39 kg-N capita^−1^ year^−1^ (10.82 kg-N capita^−1^ year^−1^ was the national average), and the food P footprint was 1.46, 1.58, 1.04. and 1.58 kg-P capita^−1^ year^−1^ (1.48 kg-P capita^−1^ year^−1^ was the national average). The findings highlight the impact of individual choice on the N and P food footprints, and the importance of encouraging the followers of religion to follow a diet consistent with the food culture of that religion. The results of this study are a clear indication of the requirement for religion-sensitive analyses in the collecting of data pertinent to a particular country for use in making government policies designed to improve the recycling of food waste and the treatment of wastewater.

## 1. Introduction

Along with potassium (K), nitrogen (N) and phosphorus (P) are essential nutrients for all living organisms. The United Nations has reported an increase in the use of both N and P as fertilizers [1]. N and P inputs as fertilizers used in the production of food are substantially higher than the N and P constituents of the food consumed [2]. Reactive N, expressed as Nr (N species apart from N gas), in air, soil and water are essential for the growth of living things [3,4]. P is also a non-substitutable nutrient required for cell division in the growth of living things. Excessive N and P in air and water is known to result in both environmental and health problems. N pollutants in the air released by burning fossil fuels can cause respiratory problems among the general public, and are one of the major contributors to acid rain and ozone layer depletion. Excessive P in the water results in eutrophication and algal blooms, which have been shown to have a growth-limiting effect on diverse ecosystems [5,6]. The toxins produced by several algal species have been shown to result in the death of aquatic life [7].

A ‘footprint’ is a parameter for the quantitative valuation of the pressure imposed on environmental resources by human beings. To effectively manage N and P along the food supply chain, it is necessary to assess the national food footprint based on the production and consumption of food items. The N footprint explains anthropogenic N loss [8], while the P footprint includes both the P resource requirement and P loss to the environment [9,10]. The N use efficiency (NUE) or P use efficiency (PUE) in food is determined by assessing how much of the N/P input reaches the final output, the food product. The N footprint is commonly determined by a country-specific bottom-up approach, known as the N-Calculator (e.g., [2,8,11,12,13]). In the case of the P footprint, the most commonly used method for country-based analysis is the material flow analysis [14,15,16,17,18]. To develop a detailed scenario of national food N and P footprints, similar system boundaries, datasets, and time periods were applied to the N-Calculator and the P-Calculator approaches [2]. While the existing N-Calculator and P-Calculator methods are based on the average per capita consumption of a set of given food items, since the consumption of many food items is subject to religious laws and rules, it is assumed that the consumption of individual food items differs considerably among religious communities.

**Figure 1:**
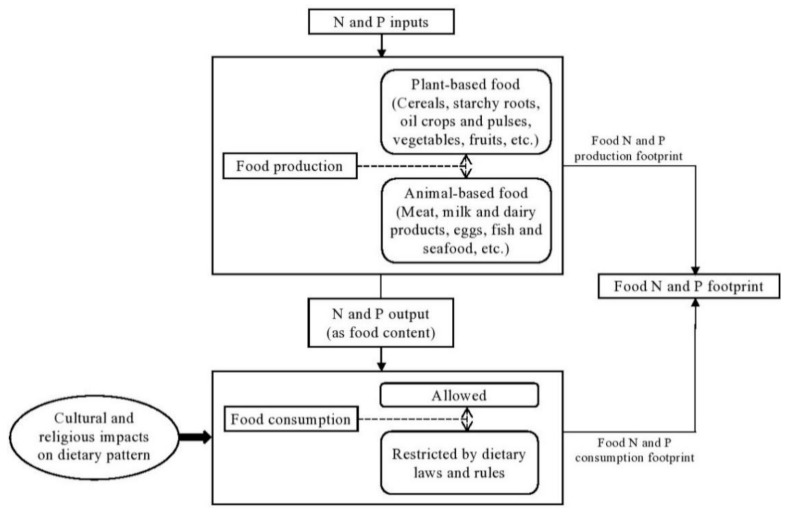
Religious dietary culture affecting food nitrogen and phosphorus footprints.

The correlation between ethnicity and nutrition can be simply expressed in terms of the food N and P footprints, as is shown in Figure 1. Food habits are determined by multiple stimuli pertaining to the dietary framework of culture and religion. Culture entails values, traditions, habits and customs, while religion represents an amalgam of cultures and beliefs. It is not always easy to distinguish between culture and religion: indeed, religion is often seen as a cultural system [19]. Food has symbolic significance in each religion and is used as a measure of respect. Therefore, the adoption of particular food patterns is in itself an expression of different religious views and an indication of whether or not the cultural and religious directives of food are being followed. Dietary habits among followers of religions often include food exclusions or taboos. For example, in Islam, eating the meat of domesticated animal species except pork is allowed, but the animal must be slaughtered using specified ‘*Halal*’ methods governed by religious laws. Most of the followers of the nonviolent Hindu and Buddhist faiths are strict vegetarians, while the Christian faith does not have any rules regarding food choice [20].

Considering this diversity among religious groups, it is reasonable to assume that the consumption of N and P from food items and the associated N and P loss to the environment varies considerably. Therefore, assessing the effects of food production and consumption of the various religious communities on N and P loss is an important step towards efficient and effective N and P management. Identifying the cultural and religious factors, and determining whether those factors have a direct or indirect impact on food production and consumption, is important for policymakers in their efforts to lower N and P footprints through policy measures. This knowledge can also be used in efforts to achieve the specific targets set by the United Nations to reduce food waste, reduce marine pollution from land-based activities and achieve food security as part of their sustainable development goals (SDGs). The country with the most diversity among its population in terms of religion is India [21]. With a total population of 1.25 billion in 2013, 79.5% are Hindus, 14.4% are Muslims, 2.5% are Christians and 0.8% are Buddhists. Only 2.8% of the population does not align itself with these religions [22,23]. The aim of this study is to explain the impact of the food consumption patterns of these religions on the N and P footprints in India. In this study, food N and P footprints are considered in terms of their association with the cultural and religious aspects of diet. For this purpose, religion-sensitive N-Calculator and P-Calculator approaches are determined. These novel N and P footprint calculation methods are applied to India.

## 2. Materials and Methods

The study is organized into two parts. In the first part, religion-sensitive N and P footprint models were developed for the N-Calculator and P-Calculator methods. In the last section, the religion-sensitive N and P footprint models were applied to India.

### 2.1. The Religion-Sensitive N-Calculator and P-Calculator Methods

In this study, the food consumption cultures of different religious groups were considered to calculate the food N and P footprints in India using a modification of the framework presented by Oita et al. (2020) [2]. The religion-sensitive N-Calculator and P-Calculator derived for this study is a population-based weighted average approach.

The dietary laws and rules of each of the four religions were considered to categorize the food habits of followers of those religions. Since only plant-based and dairy products are allowed in the Hindu faith, Hindus were regarded as lacto-vegetarians. Since Buddhism restricts the diet of its followers to plant-based products, Buddhists were classified as vegetarians. Both Muslims and Christians were classified as non-vegetarians since there are no category-wide food restrictions in these religions. Pork is strictly prohibited in Islam, and alcohol is forbidden in Hinduism, Islam and Buddhism. We categorized the food items on an FAO Food Balance Sheet [22] (Tables S1 and S2) according to the N and P intake from food by the religious groups in 2013 following dietary restrictions [20,24].

The following bottom-up approach equations of the N-Calculator method were used to estimate the food N footprint in India in 2013:

(1) $$\;\mathrm{NF}\_\mathrm{Tot}=\sum_{i=1}^{l}\sum_{j=1}^{m}W_{j}{(\mathrm{NF}\_\mathrm{Prod}}_{ij})+\sum_{i=1}^{l}\sum_{j=1}^{m}W_{j}{(\mathrm{NF}\_\mathrm{Cons}}_{ij}),$$

(2) $$\;{\mathrm{NF}\_\mathrm{Prod}}_{ij}{=\mathrm{Prot}\_\mathrm{Sup}}_{ij}{\;\times \;\mathrm{Prot}\_\mathrm{Ncont}}_{i}\;\times \;\left({1\;-\;\mathrm{Fd}\_\mathrm{Wst}}_{i}\right){\;\times \;\mathrm{VNF}\_\mathrm{Trade}}_{i},$$

(3) $${\mathrm{NF}\_\mathrm{Cons}}_{ij}{=\mathrm{Prot}\_\mathrm{Sup}}_{ij}{\;\times \;\mathrm{Prot}\_\mathrm{Ncont}}_{i}\;\times \;\left({1\;-\;\mathrm{Fd}\_\mathrm{Wst}}_{i}\right),$$

(4) $${\;\mathrm{VNF}\_\mathrm{Trade}}_{i}=\;{\mathrm{SSR}}_{i}\;\times \;{\mathrm{VNF}\_\mathrm{Dom}}_{i}{+(1\;-\;\mathrm{SSR}}_{i})\;\times \;\frac{1}{6}\sum_{x=1}^{6}{\mathrm{VNF}\_\mathrm{Dom}}_{ix},$$

(5) $${\mathrm{SSR}}_{i}=\;\frac{{\mathrm{Prod}}_{i}}{{\mathrm{Prod}}_{i}{+\mathrm{Imp}}_{i}\;{-\;\mathrm{Exp}}_{i}},$$

(6) $${\mathrm{VNF}\_\mathrm{Dom}}_{i}=\frac{{\mathrm{Nr}\_\mathrm{Loss}}_{i}}{{N\_\mathrm{Cons}}_{i}},$$

where NF_Tot is the total food N footprint, l (=94) is the total number of food items, m (=5) is the number of religious communities, *W_j_* is the population ratio of religious community *j*, NF_Prod*_ij_* is the N footprint for the production of food item *i* for religious community *j*, NF_Cons*_ij_* is the N footprint for the consumption of food item *i* for religious community *j*, Prot_Sup*_ij_* is the per capita supply of protein from food item *i* for religious community *j* [25], Prot_Ncont*_i_* is the N content of the protein supplied by food item *i* [26], Fd_Wst*_i_* is the food waste ratio of food item *i* [13,27], VNF_Trade*_i_* is the annual trade considered virtual N factor (i.e., Nr loss per unit N intake) of food item *i*, SSR*_i_* is the self-sufficiency ratio for food item *i* [25], VNF_Dom*_i_* is the annual domestic virtual N factor of food item *i*, VNF_Dom*_ix_* is the annual domestic virtual N factor of food item *i* in *x* country of the Indian subcontinent (which includes India, Bangladesh, Pakistan, Sri Lanka, Nepal and Bhutan) (Table S3), Prod*_i_* is the production quantity of food item *i* in weight, Imp*_i_* is the imported quantity of food item *i* in weight, Exp*_i_* is the exported quantity of food item *i* in weight, Nr_Loss*_i_* is the amount of Nr lost to the environment during the production of food item *i* (from N input to final consumers, the loss includes consumer-level loss), and N_Cons*_i_* is the amount of N consumed from food item *i* (N in eaten food).

Within the same system boundary, the following equations of the P-Calculator method were used to estimate the food P footprint in India in 2013:

(7) $$\;\mathrm{PF}\_\mathrm{Tot}=\sum_{i=1}^{l}\sum_{j=1}^{m}W_{j}{(\mathrm{PF}\_\mathrm{Prod}}_{ij})+\sum_{i=1}^{l}\sum_{j=1}^{m}W_{j}{(\mathrm{PF}\_\mathrm{Cons}}_{ij}),$$

(8) $${\mathrm{PF}\_\mathrm{Prod}}_{ij}{=\mathrm{Fd}\_\mathrm{Sup}}_{ij}{\;\times \;\mathrm{Fd}\_\mathrm{Pcont}}_{i}\;\times \;\left({1\;-\;\mathrm{Fd}\_\mathrm{Wst}}_{i}\right){\;\times \;\mathrm{VNF}\_\mathrm{Trade}}_{i},$$

(9) $${\mathrm{PF}\_\mathrm{Cons}}_{ij}{=\mathrm{Fd}\_\mathrm{Sup}}_{ij}{\;\times \;\mathrm{Fd}\_\mathrm{Pcont}}_{i}\;\times \;\left({1\;-\;\mathrm{Fd}\_\mathrm{Wst}}_{i}\right),$$

(10) $${\mathrm{VPF}\_\mathrm{Trade}}_{i}=\;{\mathrm{SSR}}_{i}\;\times \;{\mathrm{VPF}\_\mathrm{Dom}}_{i}{+(1\;-\;\mathrm{SSR}}_{i})\;\times \;\frac{1}{6}\sum_{x=1}^{6}{\mathrm{VPF}\_\mathrm{Dom}}_{ix},$$

(11) $${\mathrm{VPF}\_\mathrm{Dom}}_{i}=\frac{{P\_\mathrm{Loss}}_{i}}{{P\_\mathrm{Cons}}_{i}},$$

where PF_Tot is the total food P footprint, l (=94) is the total number of food items, m (=5) is the number of religious communities, *W_j_* is the population ratio of religious community *j*, PF_Prod*_ij_* is the production P footprint of food item *i* for religious community *j*, PF_Cons*_ij_* is the consumption P footprint of food item *i* for religious community *j*, Fd_Sup*_ij_* is the per capita supply of food item *i* for religious community *j* [25], Fd_Pcont*_i_* is the P content of food item *i* [26], Fd_Wst*_i_* is the food waste ratio of food item *i* [13,27], VPF_Trade*_i_* is the annual trade considered virtual P factor (i.e., P loss per unit P intake) of food item *i*, VPF_Dom*_i_* is the annual domestic virtual N factor of food item *i*, VPF_Dom*_ix_* is the annual domestic virtual P factor of food item *i* in *x* country of the Indian subcontinent (Table S3), P_Loss*_i_* is the amount of P lost to the environment during the production of food item *i* (from P input to final consumers, the loss includes consumer-level loss), and P_Cons*_i_* is the amount of P consumed from food item *i* (P in eaten food).

Since N is a major component of food protein [28], NF_Tot was estimated using the data for Prot_Sup*_ij_* and Prot_Ncont*_i_*, while PF_Tot was estimated using the data for Fd_Sup*_ij_* and Fd_Pcont*_i_* following the same framework established by Oita et al. (2020) [2].

### 2.2. Estimation of Food Waste Contributing to Total Food Nitrogen and Phosphorus Footprints

The total contribution of consumer-level food waste to the total food N footprint in India was calculated as follows:

(12) $${\;\mathrm{NF}}_{\%}\_\mathrm{DomFdWst}\_\mathrm{DomTot}=\frac{\mathrm{NF}\_\mathrm{DomFdWst}}{\mathrm{NF}\_\mathrm{DomTot}},$$

(13) $$\;\mathrm{NF}\_\mathrm{DomFdWst}=\;{\mathrm{NrLoss}\_\mathrm{Prod}}_{z}{+\mathrm{NrLoss}\_\mathrm{Cons}}_{z},$$

(14) $${\;\mathrm{NrLoss}\_\mathrm{Prod}}_{z}=\sum_{z=1}^{h}\left[{N\_\mathrm{FdSup}}_{z}{\;\times \;\mathrm{FdWst}}_{\%}{\_\mathrm{FdSup}}_{z}{\;\times \;\mathrm{VNF}\_\mathrm{DomFdWst}}_{z}\;\times \;\left({\mathrm{VNF}\_\mathrm{Dom}}_{z}\;-\;{\mathrm{VNF}\_\mathrm{DomFdWst}}_{z}\right)\right],$$

(15) $${\;\mathrm{NrLoss}\_\mathrm{Cons}}_{z}=\sum_{z=1}^{h}\left({N\_\mathrm{FdSup}}_{z}{\;\times \;\mathrm{FdWst}}_{\%}{\_\mathrm{FdSup}}_{z}{\;\times \;\mathrm{VNF}\_\mathrm{DomFdWst}}_{z}\right),$$

where NF_%__DomFdWst_DomTot is the percentage of total consumer-level food waste’s contribution to the total domestic food N footprint, NF_DomTot is the total domestic food N footprint, NF_DomFdWst is the total Nr loss driven by consumer-level food waste at producer and consumer-level, h (=33) is the total number of aggregated food items, NrLoss_Prod*_z_* is the Nr loss from waste in production of food item *z*, NrLoss_Cons*_z_* is the Nr loss from waste in consumer-level consumption of food item *z*, N_FdSup*_z_* is the N content of supplied food item *z*, FdWst_%__FdSup*_z_* is the percentage of waste to supplied food item *z*, VNF_DomFdWst*_z_* is the consumer-level wastage part of the annual domestic virtual N factor of the wasted food item *z*, and VNF_Dom*_z_* is the annual domestic virtual N factor of food item *z*.

Similarly, the total contribution of consumer-level food waste to the total food P footprint was estimated using the following equations:

(16) $${\mathrm{PF}}_{\%}\_\mathrm{DomFdWst}\_\mathrm{DomTot}=\frac{\mathrm{PF}\_\mathrm{DomFdWst}}{\mathrm{PF}\_\mathrm{DomTot}},$$

(17) $$\;\mathrm{PF}\_\mathrm{DomFdWst}=\;{\mathrm{PLoss}\_\mathrm{Prod}}_{z}{+\mathrm{PLoss}\_\mathrm{Cons}}_{z},$$

(18) $$\;\;{\mathrm{PLoss}\_\mathrm{Prod}}_{z}=\sum_{z=1}^{h}\left[{P\_\mathrm{FdSup}}_{z}{\;\times \;\mathrm{FdWst}}_{\%}{\_\mathrm{FdSup}}_{z}{\;\times \;\mathrm{VPF}\_\mathrm{DomFdWst}}_{z}\;\times \;\left({\mathrm{VPF}\_\mathrm{Dom}}_{z}\;-\;{\mathrm{VPF}\_\mathrm{DomFdWst}}_{z}\right)\right],$$

(19) $${\;\mathrm{PLoss}\_\mathrm{Cons}}_{z}=\sum_{z=1}^{h}\left({P\_\mathrm{FdSup}}_{z}{\;\times \;\mathrm{FdWst}}_{\%}{\_\mathrm{FdSup}}_{z}{\;\times \;\mathrm{VPF}\_\mathrm{DomFdWst}}_{z}\right),$$

where PF_%__DomFdWst_DomTot is the percentage of total consumer-level food waste’s contribution to the total domestic food P footprint, PF_DomTot is the total domestic food P footprint, PF_DomFdWst is the total P loss driven by consumer-level food waste at producer and consumer-level, h (=33) is the total number of aggregated food items, PLoss_Prod*_z_* is the P loss from waste in production of food item *z*, PLoss_Cons*_z_* is the P loss from waste in consumer-level consumption of food item *z*, P_FdSup*_z_* is the P content of supplied food item *z*, FdWst_%__FdSup*_z_* is the percentage of waste to purchased food item *z*, VPF_DomFdWst*_z_* is the consumer-level wastage part of the annual domestic virtual P factor of the wasted food item *z*, and VPF_Dom*_z_* is the annual domestic virtual P factor of food item *z*.

## 3. Results

**Figure 2:**
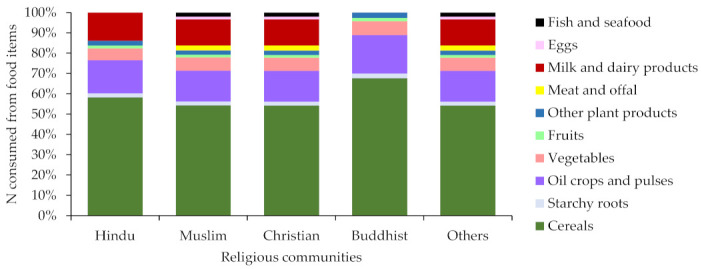
Nitrogen consumed from food by religion in India in 2013.

**Figure 3:**
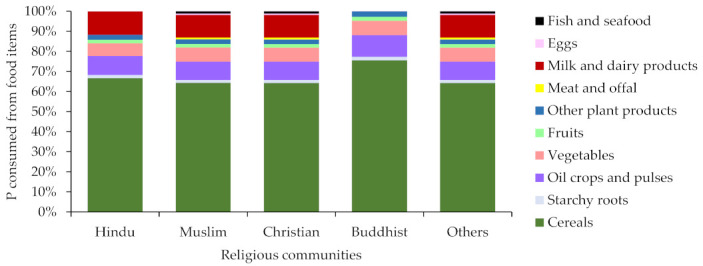
Phosphorus consumed from food by religion in India in 2013.

It has been estimated that the total food consumption in India was 603.6 Tg-food year^−1^ in 2013. The dependency on plant-based food was found to be high for all religious communities. In 2013, the total N in the consumed food was 4665 Gg-N year^−1^ and that for P was 844 Gg-P year^−1^. The VNFs of India ranged from 0.42 to 5.43 and the VPFs ranged from 0.41 to 8.15 (Table 1). The highest value for VNF was found for ‘milk and dairy products’, and ‘meat and offal’ for VPF whereas for both, the lowest value was found for starchy roots. This means that 0.42−5.43 unit Nr and 0.41−8.15 unit P would be lost to the environment for each unit of respective N and P intake from food. The results indicate a lower NUE and PUE in food production in India (the NUE was found to be 27.1% and the PUE was 42.4%). The consumed N and P was the highest in cereals. The food categories with the highest consumed N and P content are shown in Figure 2; Figure 3. Since the religious dictates in India forbid the consumption of animal flesh, the amount of N and P consumed from meat and offal was much lower than the amount from other food items. Most of the N and P consumed by vegetarian Buddhists and lacto-vegetarian Hindus was from cereal crops.

The total food N footprint in India in 2013 was estimated at 13548 Gg-N year^−1^ and in the case of P, it was estimated at 1852 Gg-P year^−1^. Since the majority of the population identify as Hindus, the total food N and P footprints of this group were the highest among the four religions (10648 Gg-N year^−1^ and 1452 Gg-P year^−1^, respectively), followed by Muslims, Christians and then Buddhists. As shown in Table 2, the food N footprints for Hindus, Muslims, Christians and Buddhists were estimated at 10.70, 11.45, 11.47 and 7.39 kg-N capita^−1^ year^−1^, and the food P footprints were estimated at 1.46, 1.58, 1.04 and 1.58 kg-P capita^−1^ year^−1^, respectively. The average per capita food N footprint in India was estimated at 10.82 kg-N capita^−1^ year^−1^ and that for P was 1.48 kg-P capita^−1^ year^−1^. Nr loss at a consumer-level food N level was 1.3% for domestically-produced food in 2013, and Nr loss due to waste in food production accounted for another 2.4%. The consumer-level P loss for domestically-produced food was 2.2%, and P loss due to waste in food production was 1.7%. Thus, consumer-level food waste was responsible for 3.7% of the food N footprint (i.e., 0.65 kg-N capita^−1^ year^−1^) and 3.9% of the food P footprint (i.e., 0.07 kg-P capita^−1^ year^−1^) of domestically-produced food in India.

## 4. Discussion

### 4.1. Factors Determining Food Nitrogen and Phosphorus Footprints

The major determinants of a country’s N and P footprints revealed in earlier studies on national level N and P scenarios are presented in Table 3 and Table 4. The major factors listed in these tables are characterized as either behavioral, technical or socioeconomic, in order to assess the influence of culture and religion on N and P footprints.

#### 4.1.1. Behavioral Factors

Because personality, circumstance and response to the environment impact behavior, individuals are capable of modifying the factors. The behavioral factors which impact the N and P footprints of the food an individual consumes include the choice to consume animal or plant-based food products, to eat a balanced diet, to waste food and to be particular about what to eat.

Leach et al. (2012) [8] found that most of the N loss to the environment related to food incurred at the production stage, and that some loss was also incurred after the food was consumed. These findings have been confirmed in many N footprint studies [2,11,28,29]. In countries with Western food culture, red meat has the highest food N footprint [8,11,28,29]. Stevens et al. (2018) [29] reported a significant change in the British food diet from 1970, when the consumption of red meat, offal and eggs was high, to 2007, when poultry meat, milk, cheese, cereals, fruits and vegetables were consumed in much greater quantities. Food waste [13,30] and unnecessary protein supply [30] have a considerable impact on the food N footprint of a nation.

The overview of the change in the global P footprint presented by Metson et al. (2012) [10] showed that the global P footprint increased by 38% in the period between 1961 and 2007. The consumption of animal-derived foods like red meat, eggs, and dairy products accounted for nearly 72% of the global P footprint. Beef was shown to contribute to almost half of the per capita P footprint from animal product consumption in the United States [40,41]. Clearly, a shift from a diet based on animal protein to a plant-based diet would result in a significant decrease in the individual P footprint at the national level.

#### 4.1.2. Technical Factors

In a recent study, it was shown that 88% of the N footprint in Tanzania was due to N loss from food production and consumption [34]. In Australia, on the other hand, the main factor was found to be intensive livestock grazing [28]. The core drivers in the almost 68% increase in the N footprint in China from 1980 to 2008 were an increase in the consumption of animal-derived food, food waste, the use of N fertilizers in crop production, livestock production, a reduction in the consumption of plant-derived food and a lower livestock manure N recycling rate [13,33]. Along with an efficient livestock system, Oita et al. (2020) [2] pointed out the importance of increasing the NUE and PUE for producing cereals and vegetables to reduce the N and P footprints. The capture and processing of wastewater were also shown to be an important factor in the increase of NUE [2,33,34].

Most of the P resources in most countries are used as fertilizers in the agriculture and fishery sectors [16,44]. In a study on the P-flow in Brazil, it was reported that the poor management of harvesting, processing and transportation of food products was responsible for a great amount of P loss [18]. Wang et al. (2011) [9] reported a PUE of only 18% in China, with the highest PUE in the food processing sector and the lowest PUE in the animal production sector. It is predicted that within 30–50 years, the P rock reserves in China will be depleted unless immediate action is taken to increase the P recycling rate [42,50,51]. Matsubae et al. (2012) [43] showed that the recycling of P has the potential to reduce Japan’s dependence on P imports.

#### 4.1.3. Socioeconomic Factors

Socioeconomic factors relate to and impact each other, and define the status of an individual on a sociological-economic scale. In the case of the N and P footprints of a country, the socioeconomic factors include the international trade of food, ammonia-derived products, P products, population growth, rural-urban migration, urbanization, gender and age differences.

In their bottom-up study on the Japanese food N footprint, Shibata et al. (2014) [12] demonstrated that the trade-considered Japanese food N footprint was lower than that of the no-trade condition due to the lower VNFs of imported foods. The estimates reported by Shindo and Yanagawa (2017) [38] in their top-down method were lower than the estimates reported by Shibata et al. (2014) [12]. Oita et al. (2016) [36] revealed that approximately 26% of the global N footprint came from the commodities traded between countries, and that the N footprint of developing countries were much lower than those of developed countries. In another study, Shibata et al. (2014) [12] found that the food N footprint of younger people in Japan was higher than that of older people, since young people tend to consume more meat and less fish. In addition, Hayashi et al. (2018) [30] found a larger food N footprint for males in every age class.

To meet their national demand for P resources, Asian countries tend to be heavily dependent on imports [47,48]. Smit et al. (2015) [14] reported that the import of fertilizer was decreasing but that the import of food and feed additives was increasing. Population growth, rural-urban migration and urbanization were the main drivers for the higher P footprint in China [32,35,46]. Along with urbanization, population growth and economic growth have been reported as the dominant factors expected to result in a higher P demand over time in Asia [47,48].

### 4.2. Association of the Identified Factors with Cultural and Religious Food Perspectives

In the existing studies, it has been acknowledged that many of the factors impacting N and P food footprints are related to culture and religion. These include such behavioral factors as the preference for animal-derived food and the treatment of food waste, technical factors in the production and grazing of livestock and treatment of wastewater and such socioeconomic factors as population growth and gender difference. While food culture is not entirely dictated by religious dictates, the preference for animal-derived food and food waste is often closely related to cultural and religious considerations. To some extent, livestock production and grazing, as well as wastewater treatment, are culturally sensitive and reflect religious viewpoints. Even though animal-based food consumption is banned in some religions and certain slaughtering methods are not allowed in other religions, the rearing of livestock as domestic animals for the purpose of producing profit is considered acceptable. Also, as long as wastewater can be treated according to religious strictures, it can be reused by members of all religions. That is, the socioeconomic factors which impact the food N and P footprints of a country, like population growth and gender difference, are influenced by religious regulations and cultural superstitions.

The cultural and religious patterns of food consumption are among the most prominent drivers of global environmental sustainability. The anthropogenic greenhouse gas (GHG) emissions associated with a vegetarian diet have been shown to be lower than those associated with a non-vegetarian diet, with those associated with the production of red meat being a major contributor [52,53]. Cultural aspects of food and diet in conjunction with religious rituals are known to contribute to the problem of food waste. For instance, nearly 30−50% of the food prepared in some Arabic countries during Ramadan is wasted due to the over-preparation of meals during this period [54]. Since this is not related to a religious dictate, it can be considered a cultural problem. Likewise in India, a total of 8 Tg-waste year^−1^ is produced from the temple, mosque and church offerings [55]. The offerings typically include milk, fruits and sweets along with flowers and tree leaves. Rather than dispose of these sacred offerings in landfills, they are thrown into rivers, ponds and lakes, and consequently pose a serious threat to the water ecosystem. Among the followers of some religions, the diet for adult males and females in the family differs [56,57,58]. Gender discrimination is another cultural aspect which affords males more nutrient-rich foods, since males are considered more valuable due to their earning potential. The use of contraceptives is also considered to be contraband in some religions. The Vatican precludes the use of contraceptives for Catholics, and followers of Islam believe they should have as many children as they may be blessed with [59,60]. In this way, the combined effects of sociocultural religious concerns result in the increased production and consumption of food, ultimately amplifying the N and P footprints of a country.

### 4.3. Comparing Findings with Other Asian Countries

The food N and P footprints in India estimated in this study are slightly lower than those reported by Oita et al. (2020) [2]. While the population ratio according to religion was considered in this study, along with the different food consumption characteristics of particular religions, the focus of the study by Oita et al. (2020) [2] was on Indian average per capita consumption of a set of given food items and the population ratios were not taken into consideration. The estimated food N and P footprints in India in both studies are significantly lower than those of China and Japan. The recent food N and P footprints in China have been estimated at 19–22 kg-N capita^−1^ year^−1^ [2,13,33] and 3–5 kg-P capita^−1^ year^−1^ [2,10]; whereas those of Japan have been estimated at 15–28 kg-N capita^−1^ year^−1^ [2,12,30,31,38] and 3–6 kg-P capita^−1^ year^−1^ [2,10]. It is clear that the religious dictate forbidding the consumption of animal-derived foods, which most of the Indian population follow, is responsible for the much lower personal food N and P footprints in India.

### 4.4. Towards a Sustainable Diet with Low Nitrogen and Phosphorus Footprints

From the findings in this study, it is reasonable to argue that the promotion of ethical and spiritual issues among particular religious communities may allow for greater control of N and P footprints. Sustainable and equitable socioenvironmental development depends on the control of these footprints, as pointed out by Oita et al. (2020) [2]. While it is ultimately important for each and every individual in the world to adopt a diet with lower N and P footprints, creating a change in dietary habits is challenging. Therefore, other measures are also needed to control the N and P footprints. Based on the findings in this study, the following measures are recommended: (i) decreasing Nr and P loss in food production through increasing NUE and PUE; (ii) raising awareness of the importance of a low-VNF/VPF diet from an ethical perspective among religious communities; (iii) improving the recycling and reuse of food waste; and (iv) implementing state of the art wastewater treatment for the recycling of N and P.

One of the most urgent challenges in reducing the N and P footprints of food is to minimize Nr and P loss during the food production process [61]. Providing farmers with adequate knowledge of the nutritional requirements of crops and optimum fertilizer application rates would be an effective approach to making progress in the effort to increase NUE and PUE, which would reduce Nr and P loss [2]. Because such information is not made available to them and fertilizer management is weakly regulated in India [62], the farmers tend to apply excessive quantities of fertilizers in the belief that it will help to ensure a good crop yield. Strict national policies and strategy guidelines are necessary to control N and P fertilizer use [63] in crop production, particularly for the high-VNF category of fruits and the high-VPF category of oil crops and pulses. By integrating crop and animal farms, crop and animal waste can be efficiently re-used at production-level, thereby increasing the NUE and PUE [61]. To reduce the high Nr and P loss from the category of milk and dairy products at the production-level, it is necessary to increase feed use efficiency through balanced feeding [64]. A reduction in the VNFs and VPFs of these plant and animal-based foods will reduce the food N and P footprints of a nation without any alteration to an individual’s religious dietary patterns.

Another approach to reducing the food N and P footprints at the consumer-level would be to promote fasting as a means of worship, as is practiced in a number of religions. On specific days of worship, fasting is practiced by both Hindus, Muslims and Buddhists in India. Hinduism prohibits eating any animal protein even the day after fasting, believing that it would go against the ethics of fasting and that it could also create digestion problems [65,66]. Fasting at least once a week, with a vegetarian diet on the following day, would be an effective way to reduce per capita N and P footprints by not only Hindus but also Muslims and Christians. This would represent a significant reduction in the consumption of high VNF/VPF animal-based foods.

To reduce food waste, households should prepare and consume food based on exactly how much they require. During Ramadan, the fasting month, Muslim households can share excess food with those in need. Not only is this kind of charitable behavior consistent with Islamic tenets, such actions will reduce Nr and P loss from the wasted food. Also, offerings by Hindus in the temples which cannot be reused again can be converted into bio-fertilizers, which will reduce Nr and P loss and also reduce the synthetic N and P inputs [67]. Since no religion supports the wasting of food, such efforts should be well-received globally. Different faith-based organizations can play a key role in achieving sustainable environmental protection [68] through campaigns designed to encourage the adoption of a balanced diet and the reduction of food waste. 

The findings of this study emphasize the need for government and non-government stakeholders, national and international organizations, corresponding extension agents and policymakers to actively inform farming communities and consumers of the necessity of N and P management. In addition to efforts to reduce the N and P food footprints in India, it is also important to reduce N and P loss by adopting appropriate wastewater treatment methods, which will ensure that the original color, odor and taste is maintained after removing the N and P pollutants, as is required by religious strictures [69]. In order to achieve these important reductions in the N and P footprints in India, effective policies need to be implemented for waste, the management of the environment and resource management.

### 4.5. Sources of Uncertainties

There are several limitations to the religion-sensitive N-Calculator and P-Calculator approaches used in this study that need to be acknowledged. Assumptions were made to make partial adjustments to religious food considerations when calculating food N and P footprints. In this study, only four of the many religions which make up the diverse religious community in India were considered. Secondly, the simple framework adopted in this study does not allow for the numerous dietary regulations of each religion to be adequately assessed. The intra-religion classifications determined by caste or diversity in beliefs no doubt affects individual food choice. However, it was assumed that everyone who identified with a religious faith strictly follows the dietary guidelines of that faith. In reality, individuals tend to interpret the rules differently, and a more relaxed attitude towards diet among some of the population would necessarily mean that the estimates of food N and P footprints would not be accurate. That is, the availability of more detailed data would allow for greater accuracy in calculating footprints.

## 5. Conclusions

The results of the religious aspects-sensitive N and P footprint analysis adopted in this study showed that the food cultures associated with a religion have a large impact on the food choices of followers of that religion. The relatively low N and P footprints in India can be attributed to these religious-based food cultures. To achieve further reductions in the food N and P footprints in India, government regulations concerning the use of fertilizers is needed to improve the NUE and PUE in food production. Religious communities should be actively encouraged to follow religious strictures which pertain to food consumption in order to keep the N and P food footprints as low as possible due to individual food choices. Faith-based organizations can take an active role in reducing food loss, promoting a more sustainable diet and ensuring the recycling of N and P from food waste. The relatively new concept of N and P footprints among other environmental footprints are useful in that they provide decision-makers with important information for reference when formulating religious sensitive nutrient management policies. Such policies are essential to developing sustainable and equitable food habits for the future. The religion-sensitive N-Calculator and P-Calculator can be used as a tool to support nutrient management policies in countries with a diversity of religions.

## Figures and Tables

**Table 1:** Domestic virtual nitrogen and phosphorus factors (VNFs and VPFs) of India in 2013.

Aggregated Food Categories	VNFs ^a^ (kg-N Loss kg-N^−1^ Intake)	VPFs ^b^ (kg-P Loss kg-P^−1^ Intake)
Cereals	1.65	1.56
Starchy roots	0.42	0.41
Oil crops and pulses	2.08	1.85
Vegetables	2.30	0.66
Fruits	2.72	1.46
Other plant products	2.14	1.02
Meat and offal	2.88	8.15
Milk and dairy products	5.43	4.01
Eggs	2.36	6.87

Note: ^a^ VNFs are defined as Nr loss per unit N intake for each food item. ^b^ VPFs are defined as P loss per unit P intake for each food item.

**Table 2:** Food nitrogen and phosphorus footprints by religion in India in 2013.

Religions	Food N Footprint		Food P Footprint	
	Individual (kg-N Capita^−1^ Year^−1^)	Total (Gg-N Year^−1^)	Individual (kg-P Capita^−1^ Year^−1^)	Total (Gg-P Year^−1^)
Hinduism	10.70	10648	1.46	1452
Islam	11.45	2065	1.58	284
Christianity	11.47	359	1.58	49
Buddhism	7.39	74	1.04	11
Others	11.47	402	1.58	55
National average	10.82	13548	1.48	1852

**Table 3:** Factors affecting food nitrogen footprint with possible cultural and religious association.

Factor’s Dimension	Factors Identified	References	Territory	Factors’ Impact on Footprint	Cultural and Religious Influence
Behaviourial	High consumption of animal-based food products (i.e., red meat, poultry meat, dairy products, eggs and fish) and low consumption of plant-based products (i.e., cereals, vegetables, fruits, etc.)	[8]	The US	↑	D
		[8]	The Netherlands		
		[11]	The EU		
		[28]	Australia		
		[2]	China		
		[2]	India		
		[2]	Japan		
		[29]	The UK		
	Excess food waste	[13]	China	↑	I
		[30]	Japan		
	Balanced diet	[31]	Japan	↓	D
	Personal food preference	[29]	The UK	≡	NI
Technical	Increase in N fertilizer use for crop production	[13]	China	↑	NI
	Increase in N use efficiency	[2,32,33]	China	↓	NI
		[2]	India		
		[2]	Japan		
	Enhancement of livestock production	[13]	China	↑	NI
	Low livestock manure N recycling rate	[13]	China	↑	NI
	Advanced wastewater treatment	[34]	Tanzania	↓	NI
	Intensive grazing	[28]	Australia	↑	NI
Socioeconomic	Increase in urbanization	[35]	China	↑	NI
	Increase in population growth	[32]	China	↑	I
	Increase in rural-urban migration	[32]	China	↑	NI
	Gender difference (focusing on male)	[30]	Japan	↑	I
	Age difference (focusing on younger aged people)	[12]	Japan	↑	NI
	International food trade	[36]	Global	↓	NI
		[37,38]	Japan		

Note: ↑, ↓ and ≡ indicate increase, decrease and moderately constant state of footprint, respectively; and D, I and NI indicate direct, indirect and no influence, respectively.

**Table 4:** Factors affecting food phosphorus footprint with possible cultural and religious association.

Factor’s Dimension	Factors Identified	References	Territory	Factors’ Impact on Footprint	Cultural and Religious Influence
Behavioral	High consumption of animal-based food products (i.e., red meat, poultry meat, dairy products, eggs and fish) and low consumption of plant-based products (i.e., cereals, vegetables, fruits, etc.)	[39]	Australia	↑	D
		[40,41]	The US		
		[10]	Global		
Technical	Increase in P use efficiency	[2,9,42]	China	↓	NI
		[2]	India		
		[2]	Japan		
	Increase in P recycling rate	[42]	China	↓	NI
		[43]	Japan		
	Increase in P fertilizer use for livestock production	[44]	Denmark	↑	NI
		[16]	Japan		
		[45]	France		
	Increase in P fertilizer use for crop production	[16]	Japan	↑	NI
		[45]	France		
	Increase in P fertilizer use for fish production	[16]	Japan		
	Poor management of food products	[18]	Brazil		
Socioeconomic	Increase in population growth	[46]	China	↑	I
		[47,48]	Asia		
	Increase in urbanization	[46]	China	↑	NI
		[47,48]	Asia		
	International trade of P products	[49]	Bangladesh	↓	NI
		[14]	The Netherlands		
		[47,48]	Asia		

Note: ↑ indicates increase state and ↓ indicates decrease state of footprint; and D, I and NI indicate direct, indirect and no influence, respectively.

## Data Availability

The data that support the findings of this study are available from the corresponding author upon reasonable request.

## Supplementary Materials

The following are available online at https://www.mdpi.com/article/10.3390/nu13061926/s1, Table S1. Nitrogen intake from food by religious groups considering food restrictions, Table S2. Phosphorus intake from food by religious groups considering food restrictions, Table S3. Average domestic virtual nitrogen and phosphorus factors (VNFs and VPFs) of the Indian subcontinent in 2013.
