# Supplementary material for: The Effect of Religious Dietary Cultures on Food Nitrogen and Phosphorus Footprints: A Case Study of India
Source: Nutrients. 2021 Jun 3;13(6):1926. doi: 10.3390/nu13061926 (PMC8226710; doi:10.3390/nu13061926)
Supplement: Supplementary file 1 [file nutrients-13-01926-s001.zip › nutrients-1231116-supplementary.pdf]

The Effect of Religious Dietary Cultures on Food Nitrogen and Phosphorus Footprints: A Case Study of India

Aurup Ratan Dhar [1,*], Azusa Oita [2] and Kazuyo Matsubae [1]

[1] Graduate School of Environmental Studies, Tohoku University, 468-1 Aoba, Aramaki, Aoba-ku, Sendai 980-0845, Miyagi, Japan
[2] Institute for Agro-Environmental Sciences, National Agriculture and Food Research Organization, 3-1-3, Kannondai, Tsukuba 305-8604, Japan
* Correspondence: aurup.ratan.dhar.p2@dc.tohoku.ac.jp

Table S1. Nitrogen intake from food by religious groups considering food restrictions.

Food Items in FAO Food Balance Sheet	Religious Restriction on Food Consumption					N Intake from Food by Religion (kg-N capita^{-1} year^{-1})				
	Hinduism	Islam	Christianity	Buddhism	Others	Hinduism	Islam	Christianity	Buddhism	Others
Wheat and products	No	No	No	No	No	0.99	0.99	0.99	0.99	0.99
Barley and products	No	No	No	No	No	0.01	0.01	0.01	0.01	0.01
Maize and products	No	No	No	No	No	0.08	0.08	0.08	0.08	0.08
Rye and products	No	No	No	No	No	-	-	-	-	-
Oats	No	No	No	No	No	-	-	-	-	-
Millet and products	No	No	No	No	No	0.12	0.12	0.12	0.12	0.12
Sorghum and products	No	No	No	No	No	0.06	0.06	0.06	0.06	0.06
Cereals, Other	No	No	No	No	No	-	-	-	-	-
Potatoes and products	No	No	No	No	No	0.07	0.07	0.07	0.07	0.07
Cassava and products	No	No	No	No	No	-	-	-	-	-
Sweet potatoes	No	No	No	No	No	-	-	-	-	-
Roots, Other	No	No	No	No	No	-	-	-	-	-
Yams	No	No	No	No	No	-	-	-	-	-
Sugar cane	No	No	No	No	No	-	-	-	-	-
Sugar non-centrifugal	No	No	No	No	No	-	-	-	-	-
Sugar (Raw Equivalent)	No	No	No	No	No	-	-	-	-	-
Sweeteners, Other	No	No	No	No	No	-	-	-	-	-
Beans	No	No	No	No	No	0.12	0.12	0.12	0.12	0.12
Peas	No	No	No	No	No	0.05	0.05	0.05	0.05	0.05
Pulses, Other and products	No	No	No	No	No	0.37	0.37	0.37	0.37	0.37

Nuts and products	No	No	No	No	No	0.01	0.01	0.01	0.01	0.01
Soyabeans	No	No	No	No	No	0.03	0.03	0.03	0.03	0.03
Groundnuts (Shelled Eq)	No	No	No	No	No	0.01	0.01	0.01	0.01	0.01
Sunflower seed	No	No	No	No	No	-	-	-	-	-
Rape and Mustardseed	No	No	No	No	No	-	-	-	-	-
Coconuts - Incl Copra	No	No	No	No	No	0.02	0.02	0.02	0.02	0.02
Sesame seed	No	No	No	No	No	-	-	-	-	-
Palm kernels	No	No	No	No	No	-	-	-	-	-
Olives (including preserved)	No	No	No	No	No	-	-	-	-	-
Oilcrops, Other	No	No	No	No	No	-	-	-	-	-
Soyabean Oil	No	No	No	No	No	-	-	-	-	-
Groundnut Oil	No	No	No	No	No	-	-	-	-	-
Sunflowerseed Oil	No	No	No	No	No	-	-	-	-	-
Rape and Mustard Oil	No	No	No	No	No	-	-	-	-	-
Cottonseed Oil	No	No	No	No	No	-	-	-	-	-
Palmkernel Oil	No	No	No	No	No	-	-	-	-	-
Palm Oil	No	No	No	No	No	-	-	-	-	-
Coconut Oil	No	No	No	No	No	-	-	-	-	-
Sesameseed Oil	No	No	No	No	No	-	-	-	-	-
Olive Oil	No	No	No	No	No	-	-	-	-	-
Ricebran Oil	No	No	No	No	No	-	-	-	-	-
Maize Germ Oil	No	No	No	No	No	-	-	-	-	-
Oilcrops Oil, Other	No	No	No	No	No	-	-	-	-	-
Tomatoes and products	No	No	No	No	No	0.02	0.02	0.02	0.02	0.02
Onions	Yes	No	No	Yes	No	-	0.04	0.04	-	0.04
Vegetables, Other	No	No	No	No	No	0.19	0.19	0.19	0.19	0.19
Oranges, Mandarines	No	No	No	No	No	-	-	-	-	-
Lemons, Limes and products	No	No	No	No	No	-	-	-	-	-
Grapefruit and products	No	No	No	No	No	-	-	-	-	-
Citrus, Other	No	No	No	No	No	-	-	-	-	-
Bananas	No	No	No	No	No	0.02	0.02	0.02	0.02	0.02
Plantains	No	No	No	No	No	-	-	-	-	-
Apples and products	No	No	No	No	No	-	-	-	-	-

Pineapples and products	No	No	No	No	No	-	-	-	-	-
Dates	No	No	No	No	No	-	-	-	-	-
Grapes and products (excl. wine)	No	No	No	No	No	-	-	-	-	-
Fruits, Other	No	No	No	No	No	0.02	0.02	0.02	0.02	0.02
Coffee and products	No	No	No	No	No	-	-	-	-	-
Cocoa Beans and products	No	No	No	No	No	-	-	-	-	-
Tea (including mate)	No	No	No	No	No	0.01	0.01	0.01	0.01	0.01
Pepper	No	No	No	No	No	-	-	-	-	-
Pimento	No	No	No	No	No	0.02	0.02	0.02	0.02	0.02
Cloves	No	No	No	No	No	-	-	-	-	-
Spices, Other	No	No	No	No	No	0.03	0.03	0.03	0.03	0.03
Wine	Yes	Yes	No	Yes	No	-	-	-	-	-
Beer	Yes	Yes	No	Yes	No	-	-	-	-	-
Beverages, Fermented	Yes	Yes	No	Yes	No	-	-	-	-	-
Beverages, Alcoholic	Yes	Yes	No	Yes	No	-	-	-	-	-
Infant food	No	No	No	No	No	-	-	-	-	-
Bovine Meat	Yes	No	No	Yes	No	-	0.02	0.02	-	0.02
Mutton & Goat Meat	Yes	No	No	Yes	No	-	0.01	0.01	-	0.01
Pigmeat	Yes	Yes	No	Yes	No	-	0.00	0.01	-	0.01
Poultry Meat	Yes	No	No	Yes	No	-	0.04	0.04	-	0.04
Meat, Other	Yes	No	No	Yes	No	-	-	-	-	0.00
Offals, Edible	Yes	No	No	Yes	No	-	0.01	0.01	-	0.01
Fats, Animals, Raw	Yes	Yes	No	Yes	No	-	-	-	-	-
Butter, Ghee	No	No	No	Yes	No	-	-	-	-	-
Cream	No	No	No	Yes	No	-	-	-	-	-
Eggs	Yes	No	No	Yes	No	-	0.05	0.05	-	0.05
Honey	No	No	No	No	No	-	-	-	-	-
Freshwater Fish	Yes	No	No	Yes	No	-	0.06	0.06	-	0.06
Demersal Fish	Yes	No	No	Yes	No	-	-	-	-	-
Pelagic Fish	Yes	No	No	Yes	No	-	0.01	0.01	-	0.01
Marine Fish, Other	Yes	No	No	Yes	No	-	0.01	0.01	-	0.01
Crustaceans	Yes	No	No	Yes	No	-	-	-	-	-
Cephalopods	Yes	No	No	Yes	No	-	-	-	-	-

Molluscs, Other	Yes	No	No	Yes	No	-	-	-	-	-
Aquatic Animals, Others	Yes	No	No	Yes	No	-	-	-	-	-
Aquatic Plants	No	No	No	No	No	-	-	-	-	-
Fish, Body Oil	Yes	No	No	Yes	No	-	-	-	-	-
Fish, Liver Oil	Yes	No	No	Yes	No	-	-	-	-	-
Rice (Milled Equivalent)	No	No	No	No	No	0.88	0.88	0.88	0.88	0.88
Milk - Excluding Butter	No	No	No	Yes	No	0.51	0.51	0.51	-	0.51
Miscellaneous	No	No	No	No	No	-	-	-	-	-

Source: [1–3].

Table S2. Phosphorus intake from food by religious groups considering food restrictions.

**Food Items in FAO Food Balance Sheet**	**Religious Restriction on Food Consumption**					**P Intake from Food by Religion (kg-P capita^{-1} year^{-1})**				
	**Hinduism**	**Islam**	**Christianity**	**Buddhism**	**Others**	**Hinduism**	**Islam**	**Christianity**	**Buddhism**	**Others**
Wheat and products	No	No	No	No	No	0.21	0.21	0.21	0.21	0.21
Barley and products	No	No	No	No	No	-	-	-	-	-
Maize and products	No	No	No	No	No	0.02	0.02	0.02	0.02	0.02
Rye and products	No	No	No	No	No	-	-	-	-	-
Oats	No	No	No	No	No	-	-	-	-	-
Millet and products	No	No	No	No	No	0.01	0.01	0.01	0.01	0.01
Sorghum and products	No	No	No	No	No	0.01	0.01	0.01	0.01	0.01
Cereals, Other	No	No	No	No	No	-	-	-	-	-
Potatoes and products	No	No	No	No	No	0.01	0.01	0.01	0.01	0.01
Cassava and products	No	No	No	No	No	-	-	-	-	-
Sweet potatoes	No	No	No	No	No	-	-	-	-	-
Roots, Other	No	No	No	No	No	-	-	-	-	-
Yams	No	No	No	No	No	-	-	-	-	-
Sugar cane	No	No	No	No	No	-	-	-	-	-
Sugar non-centrifugal	No	No	No	No	No	-	-	-	-	-
Sugar (Raw Equivalent)	No	No	No	No	No	-	-	-	-	-
Sweeteners, Other	No	No	No	No	No	-	-	-	-	-
Beans	No	No	No	No	No	0.01	0.01	0.01	0.01	0.01
Peas	No	No	No	No	No	-	-	-	-	-
Pulses, Other and products	No	No	No	No	No	0.04	0.04	0.04	0.04	0.04

Nuts and products	No	No	No	No	No	0.01	0.01	0.01	0.01	0.01
Soyabeans	No	No	No	No	No	-	-	-	-	-
Groundnuts (Shelled Eq)	No	No	No	No	No	-	-	-	-	-
Sunflower seed	No	No	No	No	No	-	-	-	-	-
Rape and Mustardseed	No	No	No	No	No	-	-	-	-	-
Coconuts - Incl Copra	No	No	No	No	No	0.01	0.01	0.01	0.01	0.01
Sesame seed	No	No	No	No	No	-	-	-	-	-
Palm kernels	No	No	No	No	No	-	-	-	-	-
Olives (including preserved)	No	No	No	No	No	-	-	-	-	-
Oilcrops, Other	No	No	No	No	No	-	-	-	-	-
Soyabean Oil	No	No	No	No	No	-	-	-	-	-
Groundnut Oil	No	No	No	No	No	-	-	-	-	-
Sunflowerseed Oil	No	No	No	No	No	-	-	-	-	-
Rape and Mustard Oil	No	No	No	No	No	-	-	-	-	-
Cottonseed Oil	No	No	No	No	No	-	-	-	-	-
Palmkernel Oil	No	No	No	No	No	-	-	-	-	-
Palm Oil	No	No	No	No	No	-	-	-	-	-
Coconut Oil	No	No	No	No	No	-	-	-	-	-
Sesameseed Oil	No	No	No	No	No	-	-	-	-	-
Olive Oil	No	No	No	No	No	-	-	-	-	-
Ricebran Oil	No	No	No	No	No	-	-	-	-	-
Maize Germ Oil	No	No	No	No	No	-	-	-	-	-
Oilcrops Oil, Other	No	No	No	No	No	-	-	-	-	-
Tomatoes and products	No	No	No	No	No	0.01	0.01	0.01	0.01	0.01
Onions	Yes	No	No	Yes	No	-	0.01	0.01	-	0.01
Vegetables, Other	No	No	No	No	No	0.04	0.04	0.04	0.04	0.04
Oranges, Mandarins	No	No	No	No	No	-	-	-	-	-
Lemons, Limes and products	No	No	No	No	No	-	-	-	-	-
Grapefruit and products	No	No	No	No	No	-	-	-	-	-
Citrus, Other	No	No	No	No	No	-	-	-	-	-
Bananas	No	No	No	No	No	-	-	-	-	-
Plantains	No	No	No	No	No	-	-	-	-	-
Apples and products	No	No	No	No	No	-	-	-	-	-

Pineapples and products	No	No	No	No	No	-	-	-	-	-
Dates	No	No	No	No	No	-	-	-	-	-
Grapes and products (excl. wine)	No	No	No	No	No	-	-	-	-	-
Fruits, Other	No	No	No	No	No	0.01	0.01	0.01	0.01	0.01
Coffee and products	No	No	No	No	No	-	-	-	-	-
Cocoa Beans and products	No	No	No	No	No	-	-	-	-	-
Tea (including mate)	No	No	No	No	No	-	-	-	-	-
Pepper	No	No	No	No	No	-	-	-	-	-
Pimento	No	No	No	No	No	-	-	-	-	-
Cloves	No	No	No	No	No	-	-	-	-	-
Spices, Other	No	No	No	No	No	-	-	-	-	-
Wine	Yes	Yes	No	Yes	No	-	-	-	-	-
Beer	Yes	Yes	No	Yes	No	-	-	-	-	-
Beverages, Fermented	Yes	Yes	No	Yes	No	-	-	-	-	-
Beverages, Alcoholic	Yes	Yes	No	Yes	No	-	-	-	-	-
Infant food	No	No	No	No	No	-	-	-	-	-
Bovine Meat	Yes	No	No	Yes	No	-	-	-	-	-
Mutton & Goat Meat	Yes	No	No	Yes	No	-	-	-	-	-
Pigmeat	Yes	Yes	No	Yes	No	-	-	-	-	-
Poultry Meat	Yes	No	No	Yes	No	-	-	-	-	-
Meat, Other	Yes	No	No	Yes	No	-	-	-	-	-
Offals, Edible	Yes	No	No	Yes	No	-	-	-	-	-
Fats, Animals, Raw	Yes	Yes	No	Yes	No	-	-	-	-	-
Butter, Ghee	No	No	No	Yes	No	-	-	-	-	-
Cream	No	No	No	Yes	No	-	-	-	-	-
Eggs	Yes	No	No	Yes	No	-	-	-	-	-
Honey	No	No	No	No	No	-	-	-	-	-
Freshwater Fish	Yes	No	No	Yes	No	-	0.01	0.01	-	0.01
Demersal Fish	Yes	No	No	Yes	No	-	-	-	-	-
Pelagic Fish	Yes	No	No	Yes	No	-	-	-	-	-
Marine Fish, Other	Yes	No	No	Yes	No	-	-	-	-	-
Crustaceans	Yes	No	No	Yes	No	-	-	-	-	-
Cephalopods	Yes	No	No	Yes	No	-	-	-	-	-

Molluscs, Other	Yes	No	No	Yes	No	-	-	-	-	-
Aquatic Animals, Others	Yes	No	No	Yes	No	-	-	-	-	-
Aquatic Plants	No	No	No	No	No	-	-	-	-	-
Fish, Body Oil	Yes	No	No	Yes	No	-	-	-	-	-
Fish, Liver Oil	Yes	No	No	Yes	No	-	-	-	-	-
Rice (Milled Equivalent)	No	No	No	No	No	0.20	0.20	0.20	0.20	0.20
Milk - Excluding Butter	No	No	No	Yes	No	0.08	0.08	0.08	-	0.08
Miscellaneous	No	No	No	No	No	-	-	-	-	-

Source: [1–3].

Table S3. Average domestic virtual nitrogen and phosphorus factors (VNFs and VPFs) of the Indian subcontinent in 2013.

Aggregated Food Categories	VNFs (kg-N Loss kg-N^{-1} Intake)	VPFs (kg-P Loss kg-P^{-1} Intake)
Cereals	0.94	0.84
Starchy roots	0.79	1.25
Oil crops and pulses	1.77	1.95
Vegetables	2.05	1.11
Fruits	5.27	4.62
Other plant products	1.78	1.05
Meat and offal	2.70	7.13
Milk and dairy products	5.23	3.73
Eggs	2.03	4.99
Fish and seafood	1.06	1.89

References

1. Kittler, P.G.; Sucher, K.P.; Nahikian-Nelms, M. *Food and culture*; 7th ed.; Cengage Learning: Boston, The United States of America, 2016; ISBN 978-1305628052.
2. Fieldhouse, P. *Food, feasts, and faith: An encyclopedia of food culture in world religions*; ABC-CLIO, LLC: California, The United States of America, 2017; ISBN 978-1610694117.
3. FAOSTAT Food balances (old methodology and population) Available online: http://www.fao.org/faostat/en/#data/FBSH.
